# Supplementary material for: Dutch Healthcare Professionals’ Opinion on the Allocation of Responsibilities concerning Prescribing and Administering Medically Indicated Vaccines to Immunocompromised Patients
Source: Vaccines (Basel). 2023 Mar 17;11(3):686. doi: 10.3390/vaccines11030686 (PMC10058813; doi:10.3390/vaccines11030686)
Supplement: Supplementary file 1 [file vaccines-11-00686-s001.zip › vaccines-2148854-supplementary.pdf]

QUESTIONNAIRE

Questions on allocation of responsibility for recommending vaccines (questions apply to medical specialists (MSs), public health specialists (PHSs), and general practitioners (GPs))

The following questions relate to the responsibility for recommending medically indicated vaccines. Please note that the questions do NOT relate to influenza vaccination. This vaccination is part of the 'National Flu Prevention Program'.

Introduction to questions 1 and 2:

A patient is being treated by a Medical specialist because of an inflammatory disease for which immunosuppressive therapy is prescribed.

Question 1:

Who is responsible for advising medically indicated vaccines to the patient?

- o Primary treating physician
- o General practitioner

Question 2:

Who is responsible to decide which vaccines are needed in this case? (multiple answers possible)

- o Primary treating physician and/or his/her team (i.e., nurse specialist)
- o A consulted specialist in hospital (i.e., an infectiologist/rheumatologist/clinical immunologist)
- o General practitioner
- o Public health specialist

Question 3:

A number of possible experienced barriers for prescribing medically indicated vaccines are listed below. Please indicate to what extent these barriers apply to you.

Strongly agree = this is a barrier

Strongly disagree = this is not a barrier

Barrier:	Strongly Agree	Agree	Not Agree, not Disagree	Disagree	Strongly Disagree
Protocols are missing	0	0	0	0	0
Protocols are not easy to find	0	0	0	0	0
Lack of reimbursement and patients have to pay themselves	0	0	0	0	0
Arrangements on reimbursement are unclear/difficult to find	0	0	0	0	0
I don't have a good overview of the patient's vaccination history	0	0	0	0	0
I feel I have insufficient knowledge on vaccines	0	0	0	0	0
I have little confidence in vaccine effectiveness	0	0	0	0	0
My patient is already on immunosuppressants and therefore I don't think vaccination is effective	0	0	0	0	0
Many medically indicated vaccines are not indicated according to my point of view	0	0	0	0	0
I do not have enough time to discuss vaccines in my outpatient clinics	0	0	0	0	0

Questions on allocation of responsibility for administering vaccines (questions apply to MSs, PHSs, GPs)

The following questions relate to the administration of medically indicated vaccines. Please note that the questions do NOT relate to influenza vaccination. This vaccination is part of the 'National Flu Prevention Program'.

Introduction to question 4:

A patient is being treated by a Medical specialist because of an inflammatory disease for which immunosuppressive therapy is prescribed. An indication for pneumococcal vaccination has been made by the Medical specialist.

Question 4:

Where should this patient be vaccinated? (Multiple answers possible)

- In hospital, via the medical specialist and his/her team (primary treating physician incl. a nurse specialist)
- In hospital, via a consulted specialist and his/her team (e.g., infectiologist/rheumatologist/clinical immunologist incl. a nurse specialist)
- At the GP's office, on the recommendation of the medical specialist
- At the Municipal Public Health Service (MPHS)
- At the pharmacy

Additional questions for the Medical specialist

Below are a number of barriers for in hospital administration of medically indicated vaccines (i.e., by a nurse specialist under your responsibility). Please indicate to what extent these barriers apply to you.

Strongly agree = this is a barrier

Strongly disagree = this is not a barrier

Question 5:

Barriers:	Strongly Agree	Agree	Not Agree, not Disagree	Disagree	Strongly Disagree
Problems with / lack of clarity about reimbursement (leaving the hospital to bear the costs)	0	0	0	0	0
Logistical problems (no nurse for vaccine administration, insufficient availability of vaccines)	0	0	0	0	0
Not the responsibility of the Medical specialist and/or his/her treating team	0	0	0	0	0

Question 6:

Below are some arguments for in hospital administration of medically indicated vaccines (i.e., by a nurse specialist under your responsibility). You may assume that there are no restrictions. Please indicate to what extent these arguments apply to you.

Strongly agree = this is an argument for in hospital administration

Strongly disagree = this is not an argument for in hospital administration

Arguments for in Hospital Administration	Strongly Agree	Agree	Not Agree, not Disagree	Disagree	Strongly Disagree
I see the administration as my responsibility	0	0	0	0	0
I think my team has the right expertise for it	0	0	0	0	0
If I outsource it, I don't know whether my advice will be followed by the patient or GP/Public health specialist	0	0	0	0	0
I find in hospital administration more patient-friendly (in some cases)	0	0	0	0	0

Additional questions for the Public health specialist

Below are a number of barriers to administer medically indicated vaccines, prescribed by a Medical specialist, at the MPHS. Please indicate to what extent these barriers apply to you.

Strongly agree = this is a barrier

Strongly disagree = this is not a barrier

Barriers	Strongly Agree	Agree	Not Agree, not Disagree	Disagree	Strongly Disagree
Lack of expense reimbursement	0	0	0	0	0
There is no structural collaboration with MSs in the hospital (yet)	0	0	0	0	0
We have no experience in vaccination of immunocompromised patients	0	0	0	0	0
We do not have all vaccines in stock	0	0	0	0	0
We do not offer specific vaccination care for immunocompromised patients	0	0	0	0	0

Below are some arguments for administering medically indicated vaccines, prescribed by a Medical specialist, at the MPHS. You may assume that there are no restrictions. Please indicate to what extent these arguments apply to you.

Strongly agree = this is an argument for administration at MPHS

Strongly disagree = this is not an argument for administration at MPHS

Arguments for Administration at MPHS	Strongly Agree	Agree	Not Agree, not Disagree	Disagree	Strongly Disagree
I see it as a responsibility of the MPHS	0	0	0	0	0
The MPHS has a lot of knowledge about administering vaccines	0	0	0	0	0
The MPHS has a lot of experience in administering vaccines	0	0	0	0	0

Additional questions for the General Practitioner

Below are a number of barriers to administer medically indicated vaccines, prescribed by a Medical specialist, at your office. Please indicate to what extent these barriers apply to you.

Strongly agree = this is a barrier

Strongly disagree = this is not a barrier

Barriers	Strongly Agree	Agree	Not Agree, not Disagree	Disagree	Strongly Disagree
Lack of expense reimbursement	0	0	0	0	0
Lack of communication between Medical specialist and GP (e.g., no/incomplete referral letter)	0	0	0	0	0
Logistical problems in ordering and storage of vaccines	0	0	0	0	0
Not the responsibility of the GP/GP's assistant	0	0	0	0	0

Below are some arguments for administering medically indicated vaccines, prescribed by a Medical specialist, at your office. You may assume that there are no restrictions. Please indicate to what extent these arguments apply to you.

Strongly agree = this is an argument for administration at the GP

Strongly disagree = this is not an argument for administration at the GP

Arguments of Administration at the GP	Strongly Agree	Agree	Not Agree, not Disagree	Disagree	Strongly Disagree
I see it as my responsibility because I know the patient best	0	0	0	0	0
I think I have the most experience	0	0	0	0	0
I think I have the best knowledge	0	0	0	0	0
It could be more patient-friendly	0	0	0	0	0

Questions on solutions (questions apply to MSs, PHSs, GPs)

From the advisory report by the Dutch institute of health, several solutions have been put forward for problems related to vaccination care for immunocompromised patients. These solutions are listed below. Please indicate to what extent these solutions would help you to indicate and/or administer medically indicated vaccines.

Strongly agree = this would help me

Strongly disagree = this would not help me

Solutions:	Strongly Agree	Agree	Not Agree, not Disagree	Disagree	Strongly Disagree
An overarching guideline on vaccination care for medical risk groups	0	0	0	0	0
A clear protocol about practical aspects of administering vaccines *	0	0	0	0	0
Education on this topic for doctors and nurse specialists	0	0	0	0	0
Availability of patient information on this topic	0	0	0	0	0
Integrated reminders in the patient file	0	0	0	0	0
Clear and comprehensible registration of vaccinations received	0	0	0	0	0
Well-regulated reimbursement for vaccines and/or vaccination care	0	0	0	0	0
Improved logistics within my practice/hospital/organization	0	0	0	0	0
Better collaboration between healthcare providers from primary, secondary, and tertiary care	0	0	0	0	0

* For example: contraindications to vaccination, interaction with other vaccines, information on administration of multiple vaccines at the same time.
